# Nanoscale Colocalization of NK Cell Activating and Inhibitory Receptors Controls Signal Integration

**DOI:** 10.3389/fimmu.2022.868496

**Published:** 2022-06-01

**Authors:** David Tomaz, Pedro Matos Pereira, Nadia Guerra, Julian Dyson, Keith Gould, Ricardo Henriques

**Affiliations:** ^1^ Department of Immunology, Wright-Fleming Institute, Imperial College London, London, United Kingdom; ^2^ Department of Immunology, Hammersmith Hospital, Imperial College London, London, United Kingdom; ^3^ Medical Research Council Laboratory for Molecular Cell Biology, University College London, London, United Kingdom; ^4^ Bacterial Cell Biology, Instituto de Tecnologia Química e Biológica António Xavier, Universidade Nova de Lisboa, Oeiras, Portugal; ^5^ Division of Cell and Molecular Biology, Faculty of Natural Sciences, Imperial College London, London, United Kingdom; ^6^ Division of Infection and Immunity, University College London, London, United Kingdom; ^7^ Optical Cell Biology Lab, Instituto Gulbenkian de Ciência, Oeiras, Portugal

**Keywords:** NK cell signaling, nanoscale colocalization, NKG2D, Ly49A, NK cell receptors and ligands, FRET, signal integration

## Abstract

Natural killer (NK) cell responses depend on the balance of signals from inhibitory and activating receptors. However, how the integration of antagonistic signals occurs upon NK cell–target cell interaction is not fully understood. Here we provide evidence that NK cell inhibition *via* the inhibitory receptor Ly49A is dependent on its relative colocalization at the nanometer scale with the activating receptor NKG2D upon immune synapse (IS) formation. NKG2D and Ly49A signal integration and colocalization were studied using NKG2D-GFP and Ly49A-RFP-expressing primary NK cells, forming ISs with NIH3T3 target cells, with or without the expression of single-chain trimer (SCT) H2-Dd and an extended form of SCT H2-Dd-CD4 MHC-I molecules. Nanoscale colocalization was assessed by Förster resonance energy transfer between NKG2D-GFP and Ly49A-RFP and measured for each synapse. In the presence of their respective cognate ligands, NKG2D and Ly49A colocalize at the nanometer scale, leading to NK cell inhibition. However, increasing the size of the Ly49A ligand reduced the nanoscale colocalization with NKG2D, consequently impairing Ly49A-mediated inhibition. Thus, our data shows that NK cell signal integration is critically dependent on the dimensions of NK cell ligand–receptor pairs by affecting their relative nanometer-scale colocalization at the IS. Our results together suggest that the balance of NK cell signals and NK cell responses is determined by the relative nanoscale colocalization of activating and inhibitory receptors in the immune synapse.

## Introduction

Natural killer (NK) cell activation is dependent on a different array of germline-encoded receptors capable of triggering effector responses against infection (1–3) and cellular transformation (4, 5) and yet maintaining tolerance towards healthy cells and tissues (6, 7). NK cell functionality is widely characterized by a balance between activating and inhibitory receptors (8, 9). However, upon immune synapse formation, how NK cells integrate signals from functionally antagonistic receptors is not yet fully understood (10). One major hypothesis to explain immune signal integration is based on the kinetic segregation (K-S) model (11). This model states that the balance of antagonistic signals is mediated by the equilibrium of phosphorylation (kinases) and dephosphorylation (phosphatases), which depends on the relative colocalization of receptors and their associated signaling molecules upon synapse formation. This hypothesis has been validated using T cells, in the context of TCR triggering (12, 13), and, more recently, NK cells (14, 15). The K-S model suggests that immune inhibition, upon immune synapse formation, is maintained by the dephosphorylation of tyrosines in stimulatory receptors (*e*.*g*., ITAMs-associated receptors such as TCR-CD3 or NKG2D-DAP12) due to a nanoscale colocalization with receptors bearing phosphatase activity (*e*.*g*., CD45 and CD148) or with the capacity of recruiting phosphatases, such as Src homology region 2 domain-containing phosphatase-1 and -2 (SHP1/2) or phosphatidylinositol-3,4,5-trisphosphate 5-phosphatase 1 (SHIP) [*e*.*g*., immunoreceptor tyrosine-based inhibitory motifs (ITIMs)-associated receptors such as PD1, KIR2DL2, or Ly49A] (16, 17). Several recent studies have investigated the relationship between immune signal integration and the colocalization of antagonistic signal receptors. Using altered dimensions of receptor–ligand pairs, it has been shown that the molecular dimensions of receptor–ligand pairs have a proportional, size-dependent effect on immune cell signal integration and functionality in both T and NK cells. In T cells, TCR-MHC-I dimensions have been shown to play a major role in triggering TCR activation (12). The molecular dimensions of CD45 and CD148 have also been reported to affect T cell functionality (18). More recently, the initiation of T cell signaling was confirmed by CD45 segregation at close contact points (19). In NK cells, elongation of activating and inhibitory NK cell receptor ligands has been shown to affect NK cell activation and inhibition, respectively (14, 15). These studies showed that elongation of H60a reduces NKG2D-mediated activation, without reducing binding. Similarly, elongating ligands for both the inhibitory Ly49C/I and CD94/NKG2A receptors reduced NK cell inhibition (14). In another study using human NK cells, it was found that elongated versions of HLA-Cw6 and MICA reduced their inhibition and activation, respectively (15). This study also suggested that a relative colocalization of matched-size NKG2D/MICA and KIR2DL1/HLA-Cw6 complexes is required for efficient NK cell signal integration. Although multiple combinations of NK cell activating and inhibitory receptors have been identified (20–22), their relative contributions to NK cell responses need to be further investigated. A murine model study by Regunathan et al. (2005) investigated the balance of signals between Ly49A and NKG2D. This study showed that NKG2D-dependent activation could be counterbalanced by inhibitory receptors, in particular, Ly49A (23). The interplay of signaling strength between the inhibitory receptors, Ly49A/G, and the activating receptor, NKG2D, was shown to determine the magnitude of cell activation. Ly49A was effective at inhibiting NKG2D-dependent responses, even in the presence of high levels of NKG2D ligand expression (23). Ly49A-mediated inhibition is hypothesized to occur due to its relative proximity to activating receptors (*e*.*g*., NKG2D) and the proximal recruitment of (SHP1/2 or SHIP (24, 25). However, a nanometer-scale colocalization of Ly49A with activating receptors, upon immune synapse formation, in primary NK cells has not yet been reported. In the present study, we used retrogenic mice to generate primary NK cells expressing fluorescent protein (FP)-tagged Ly49A and NKG2D receptors. The colocalization of NKG2D-GFP and Ly49A-RFP was investigated on a nanometer scale by measuring the Förster resonance energy transfer (FRET) between the FP-tagged receptors upon synapse formation. Interestingly, we observed the colocalization of Ly49A and NKG2D upon IS formation. These results suggest that the balance of NK cell signals might be determined by the relative colocalization of activating and inhibitory receptors at the nanometer scale. These new findings are important as they suggest a molecular mechanism of balance of NK cell signals dependent on a differential organization of receptors upon synapse formation.

## Materials and Methods

### DNA Constructs and Fusion Proteins

Mouse NKG2D short and long isoforms (NKG2D-S/L) and Ly49A were cloned into pTagGFP or pTagRFP (Evrogen), respectively, using NKG2D and Ly49A cDNA clone expression vectors (Origene). NKG2D-S/L was generated by PCR using forward 5′-TAGTAGTCTCGAGCCACCATGAGCAAATGCCATAATTACGACCTC-3′ (short isoform, NKG2D-S) or 5′-TAGTAGTCTCGAGCCACCATGGCATTGATTCGTGATCGA-3′ (long isoform, NKG2D-L) and the reverse 5′-TAGTAGCCCCGGGCCTTACACCGCCCTTTTCATGCAG-3′ primers, with added XhoI and XmaI sites, and then cloned into the pTagGFP plasmid between the XhoI and XmaI restriction sites. Ly49A cDNA was amplified using forward 5′-TAGTAGTCTCGAGCCACCATGAGTGAGCAGGAGGTCACTTATT-3′ and reverse 5′-TAGTAGCCCCGGGCCTCAATGAGGGAATTTATCCAGTCTC-3′ primers, with added XhoI and XmaI sites, and cloned into the pTagRFP plasmid. To subclone the fusion protein construct GFP-NKG2D-S/L into the retroviral stem cell vector pMIGR1 (Addgene, plasmid 27490), forward 5′-TAGTAGGAATTCGCCACCATGAGCGGGGGCGAGGAC-3′ and reverse 5′-TAGAGGTCGACCTTACACCGCCCTTTTCATGCAG-3′ primers were used. RFP-Ly49A was subcloned into pMIGR1 using forward 5′-TAGTAGGAATTCGCCACCATGGTGTCTAAGGGC-3′ and the reverse 5′-TAGTAGGGTCGACGCTCAATGAGGGAATTTATCCAGTCTC-3′ primers, with added EcoRI and SalI restriction sites. Both GFP-NKG2D-S/L and RFP-Ly49A were subcloned into the pMIGR1 plasmid between EcoRI and SalI restriction sites. C57BL/6-derived mRNA was reverse-transcribed, and DAP10 and DAP12 encoding cDNA were amplified by PCR and subsequently cloned into the pcDNA3.1 (hyg+) plasmid using HindIII and XhoI restriction sites. DAP10 cDNA was amplified using forward 5′-TAGTAGGAAGCTTCCACCATGGACCCCCCAGGCTACCTC-3′ and 5′-TAGTAGCCTCGAGCCTCAGCCTCTGCCAGGCATGTTGAT-3′ reverse primers, while DAP12 cDNA was amplified using forward 5′-TAGTAGGAAGCTTCCACCATGGGGGCTCTGG-3′ and 5′-TAGTAGCCTCGAGCCTCATCTGTAATATTGCCTCTGTGTGTT-3′ reverse primers. DNA constructs encoding a single-chain trimer (SCT) version of H2-Dd and H2-Dd-CD4 (hereafter termed Dd and Dd-CD4, respectively) were generated in pKG4, a eukaryotic mammalian expression vector conferring geneticin resistance. The SCT Dd-CD4 elongated form of SCT Dd was generated as described in a previous study for elongated H60a molecules, using an introduced unique BamHI restriction site. The CD4 insert encoding the domains 1–4 of human CD4 (Dd-CD4) was excised and ligated into the BamHI site in the Dd cDNA as previously described for the H60a-CD4 molecule. H60a and H60a-CD4 molecules were generated previously (15). All DNA constructs and plasmid inserts were sequenced and verified at the MRC CSC Genomics Core Laboratory (Imperial College, UK).

### Cell Lines and Expression of Cell Surface Proteins

NIH3T3, CHO-K1, HEK 293T, and RMA cells were grown in IMDM, Ham’s F12, and RPMI medium, respectively, supplemented with 10% fetal calf serum (FCS) and 2 mM L-glutamine and transfected using the Lipofectamine 2000 reagent (Life Technologies), following the manufacturer’s instructions. The NIH3T3 and CHO-K1 transfectants were maintained in 0.5 mg/ml geneticin, and SCT Dd or SCT Dd-CD4 cell surface expression was regularly assessed by flow cytometry (clones 5-85 and 34-2-12, Abcam and Biolegend, respectively). The transfectants were FACS-sorted (FACSDiva and FACSARIAIIIU cell sorter, BD) for the cell surface expression of the relevant molecule, and stable transfectants were further selected using 0.8 mg/ml geneticin. Protein cell surface expression was detected by flow cytometry (FACSDIVA, BD) using anti-NKG2D (clone C7, Biolegend), anti-Ly49A (clone YE1/48.10.6, Biolegend), anti-CD3 (clone 17A2, Biolegend), anti-NK1.1 (clone PK136, Biolegend), anti-NKp46 (clone 29A1.4, Biolegend), anti-CD49b (clone DX5, Biolegend), and anti-H60a (clone 205326, R\&D systems) antibodies.

### Mouse Primary Cell Culture and NK Cell Isolation

All primary cells were cultured in RPMI 1640 medium, supplemented with 10% FCS, 2 mM L-glutamine, 20 mM HEPES buffer, sodium pyruvate 1×, MEM non-essential amino acids solution 1×, and 50 µM β-mercaptoethanol. Spleens were harvested from 4- to 16-week-old male C57BL/6 (B6) mice and Klrk1-/- B6 mice (25) (housed under standard conditions at the Imperial College St. Mary’s or Hammersmith CBS Animal Facilities). NK cells were isolated by MACS using the Mouse NK Isolation Kit II (Miltenyi Biotec, Germany), following the manufacturer’s instructions. For the enrichment of a specific NK cell subset, the cell population of interest was stained with a biotin-conjugated antibody specific for the receptor of interest and subjected to MACS using anti-biotin microbeads (Miltenyi). Isolated primary NK cells were cultured in supplemented RPMI 1640 medium and stimulated with 1,000 U/ml human recombinant IL-2 (Cetus Corporation) for 5 to 7 days before use. The generation of *in vitro* differentiated NK cells was performed using hematopoietic progenitor cells (HPCs) isolated using the mouse Lineage Cell Depletion Kit (Miltenyi Biotec). Lineage negative HPCs were transduced with either GFP-NKG2D-S/L or RFP-Ly49A and cultured in a differentiation-conditioned RPMI medium as described (26).

### Flow Cytometry-Based NK Cell Cytotoxicity and Conjugation Assays

Target cells were stained with either CellTraceTM CFSE Cell Proliferation Kit (Life Technologies) or CellTraceTM Violet Cell Proliferation Kit (Life Technologies), and 1 × 10^5^ target cells were placed in FACS tubes. Purified splenic NK cells expanded in IL-2 for 4 to 6 days were used as effector cells at various effector/target cell ratios. The cells were incubated together for 6 h at 37°C and then fixed with 4% paraformaldehyde (PFA) for 15 min. Before FACS acquisition, the cells were resuspended in phosphate-buffered saline (PBS) with 30 nM of 4′,6-diamidino-2-phenylindole (Life Technologies). In each tube, 10 μl of bright beads (Life Technologies) was added for precise cell counting, and the relative number of cells lysed was extrapolated according to the manufacturer’s instructions. For the conjugation assays, NIH3T3 and RMA target cells were labeled with a CellTrace Violet Cell Proliferation Kit (Life Technologies). After labeling, the cells were washed four times and rested for 1 h at 37°C. Equal numbers of effector cells (expressing fluorescent receptors) and target cells were mixed in a 200-μl volume and briefly centrifuged to stimulate conjugate formation. The cells were then incubated for a further 5 min and fixed using 4% PFA for 15 min. The cells were then gently resuspended in FACS buffer and analyzed by flow cytometry. The relative ratio and percentage of duplets and double positive events were recorded as estimates of conjugate formation.

### Retrogenic Mice and Primary Cell Transduction

The mice were housed under specific pathogen-free conditions at Imperial College London. All experimental protocols were approved by the Institutional Animal Welfare and Ethical Review Committee and by the Home Office and were performed in accordance with the relevant guidelines and regulations. NKG2D-S/L and Ly49A receptors fused to GFP or RFP, respectively, were generated *in vivo* and expressed in primary NK cells using retroviral transduction and the retrogenic mice technique (27). For retrogenic expression, GFP-NKG2D-S/L and RFP-Ly49A were cloned into the pMIGR1 retroviral vector and transduced into bone marrow (BM) cells, which were then injected intravenously into irradiated (600 rad) C57BL/6J recipients as described (27). Briefly, BM cells were collected from 4- to 16-week-old C57BL/6J Klrk1-/- mice (Klrk1-/-, H2-Kb) (25, 28) previously injected i.p. with 150 mg/kg 5-fluorouracil (InvivoGen) 48 to 72 h before culling. The BM cells were transduced using a spin infection protocol. In total, 5 × 10^6^ BM cells were re-suspended in 2 ml of viral supernatant with 8 µg/ml polybrene and centrifuged at 1,500 rpm for 1.5 h at room temperature. The cells were transduced once or twice, depending on the relative efficiency of transduction as analyzed by flow cytometry. The recipient mice were sub-lethally irradiated with 600 rad, and each mouse was injected in the tail vein with at least 5 × 10^6^ cells, unless stated otherwise. The retrogenic (RT) mice were bled weekly by nicking the tail vein and checked for reconstitution by flow cytometry.

### Confocal Microscopy and Image Analysis

NK cells and target cells were mixed in chamber slides, and live cell–cell conjugates were imaged at 5% CO_2_ and 37°C by confocal microscopy (Leica SP5), using a ×63 oil-immersed objective. For FRET conjugation experiments, target cells were stained with a CellTrace™ Violet Cell Proliferation Kit (Life Technologies), and primary NK cells *ex vivo* were stimulated with IL-2 in culture for 6 to 8 days. Immunological synapses were formed by incubating the target cells together with NK cells at a ratio of approximately 1:3 NK cell/target cells. The target cells were seeded onto µ-Slide 8-well glass-bottomed plates (Ibidi) 24 h before use and left in cell culture overnight at 37°C in complete RPMI medium. NK cells were added on top of the adherent monolayer of NIH3T3 target cells and centrifuged at 1,500 rpm for approximately 5 min to form synapses. The cells were then fixed in ice-cold 4% paraformaldehyde for 15 min and washed twice in PBS, and 200 µl of Mowiol was added to each well. FRET conjugate images were acquired using a Leica TCS SP5 confocal microscope (Leica DMI6000) with a ×63 oil-immersed objective. The images were processed using Fiji software (ImageJ, NIH, Bethesda, MD, USA) (29). FRET analysis was performed in accordance with the developer’s instructions using the ImageJ plugin AccPbFRET (http://www.biophys.dote.hu/accpbfret/) for analysis of acceptor photobleaching FRET images (30). For FRET analysis, images were obtained from the donor (GFP, 488 nm excitation laser) and acceptor (RFP, 561 nm excitation laser) channels before and after acceptor photobleaching. To avoid crosstalk and compensation between different excitation channels, the images were captured in sequential mode. Each synapse corresponded to a single z-stack, and all confocal images were collected using a pinhole of 1 Airy unit diameter automatically adjusted to maintain the same optical section thickness between channels.

### Statistical Analysis

Statistical analysis, including arithmetic means, standard deviations, one-way ANOVA, Mann–Whitney and Wilcoxon matched-pairs rank tests, and unpaired and multiple *t*-tests were performed using Prism 6 (GraphPad) as indicated. The results were considered statistically significant when the associated *P*-value was lower than 0.05 (*<0.05, **<0.01, ***<0.001, ****<0.0001; ns, no statistical significance, *p* > 0.05).

## Results

### Generation of NK Cells Co-expressing NKG2D-GFP and Ly49A-RFP

In this work, we created RT mice displaying NK cells expressing NKG2D-GFP and Ly49A-RFP receptors. Several previous studies have used FP-tagged NK cell receptors or ligands to study NK cell signaling. These studies expressed NKG2D- and Ly49A-tagged receptors on T and NK-like cell lines (31, 32). In this study, we created authentic primary C57BL/6 (B6) NK cells expressing FP-tagged NK cell receptors *in vivo*. The RT mice technique has previously been used to create specific TCR- and BCR-expressing T and B cells, respectively (33, 34), but here we applied it for the first time to create NK cells expressing FP-tagged receptors *in vivo* (**Supplementary Figure S1**).

**Figure 1:**
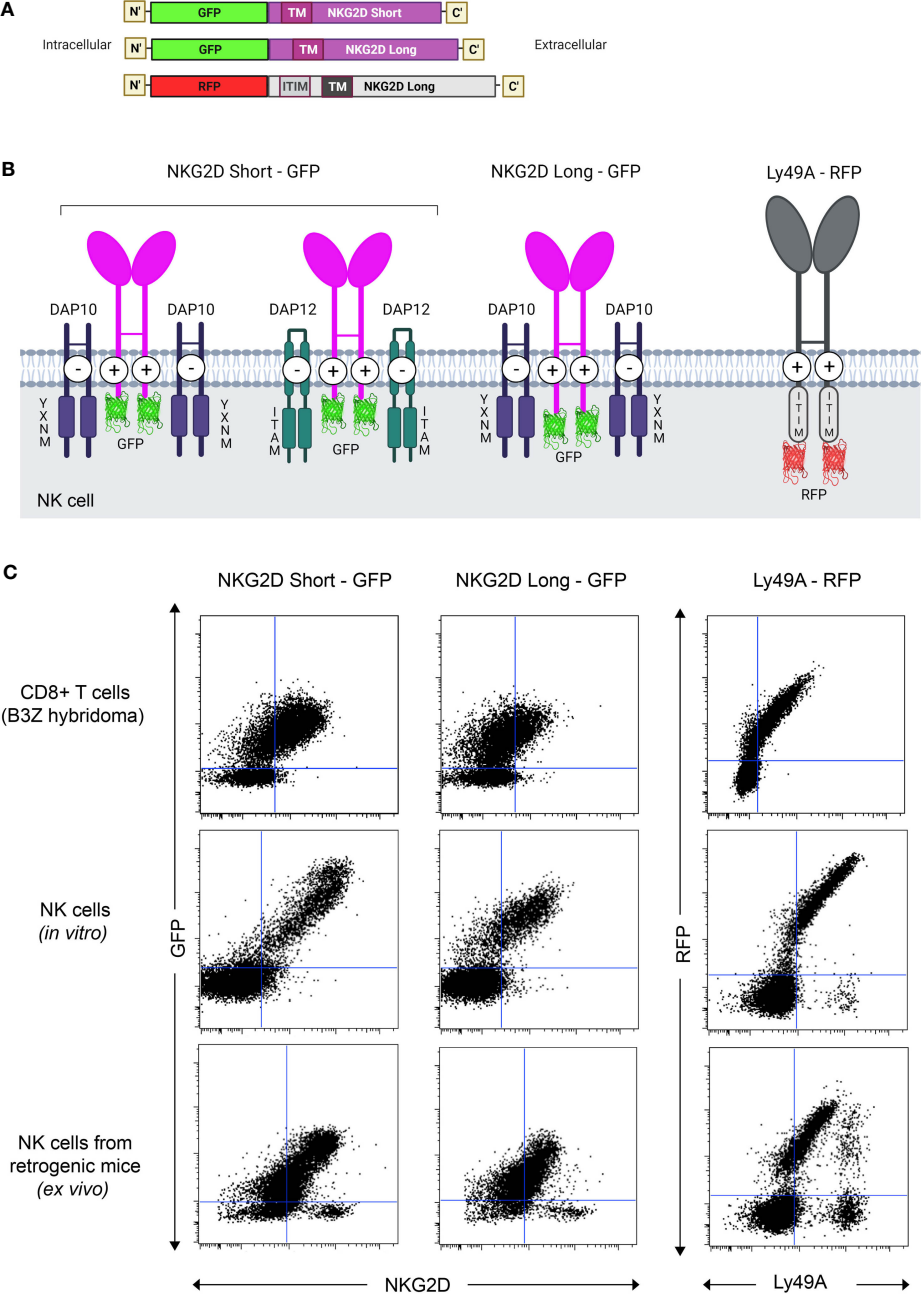
Generation and expression of NKG2D(S/L)-GFP and Ly49A-RFP-expressing natural killer (NK) cells *in vivo*. **(A)** Schematic representation of the constructs of mouse NKG2D-GFP and Ly49A-RFP used for functional and colocalization studies. All constructs were subcloned into the pMIGR1 retroviral expression vector. **(B)** Schematic of NKG2D-GFP and Ly49A-RFP at the NK cell surface. Both molecules form homodimers. Image representation is not to scale. **(C)** NK cells expressing NKG2D-GFP and Ly49A-RFP generated *in vivo*. Retroviral-mediated expression of NKG2D-GFP (S/L) and Ly49A in murine B3Z CD8+ T cell line (top), NK cells differentiated *in vitro* from hematopoietic progenitor cells (HPCs, middle) and NK cells obtained from retrogenic (RT) mice (bottom, see also **Supplementary Figure S1**). Experiments with NK cells differentiated *in vitro* were performed by transducing HPCs from NKG2D-deficient C57BL/6 mice (**Supplementary Figure S2**), and the data shown are gated on NK1.1+, NKp46+, and CD3- cells. Splenic NK cells from RT mice were gated as CD3-, CD8-, NK1.1+, and CD49+ (DX5) cells. The data are representative of more than three experiments.

pMIGR1 expressing the long and short isoforms of NKG2D-GFP (NKG2D-L-GFP and NKG2D-S-GFP) and Ly49A-RFP were generated (**Figures 1A, B**). By retroviral transduction, we expressed NKG2D-GFP and Ly49A-RFP in three different immune cells—(i) B3Z CD8+ T cell hybridoma, (ii) HPC-derived NK cells, and (iii) NK cells *in vivo*—from RT mice (**Figure 1C**). The NK cells from RT mice were generated as previously described (27, 35). In total, 293 T cells were efficiently transfected with NKG2D-GFP and Ly49A-RFP (**Supplementary Figures S2, S3**). HPCs from NKG2D-deficient mice were transduced and used as donor cells (**Supplementary Figures S4, S5**). NKG2D-deficient donor cells were used to avoid the expression of endogenous, untagged NKG2D. HPCs were first transduced and then differentiated *in vitro* into HPC-derived NK cells as previously described. B3Z CD8+ T cell hybridoma cells were directly transduced.

**Figure 2:**
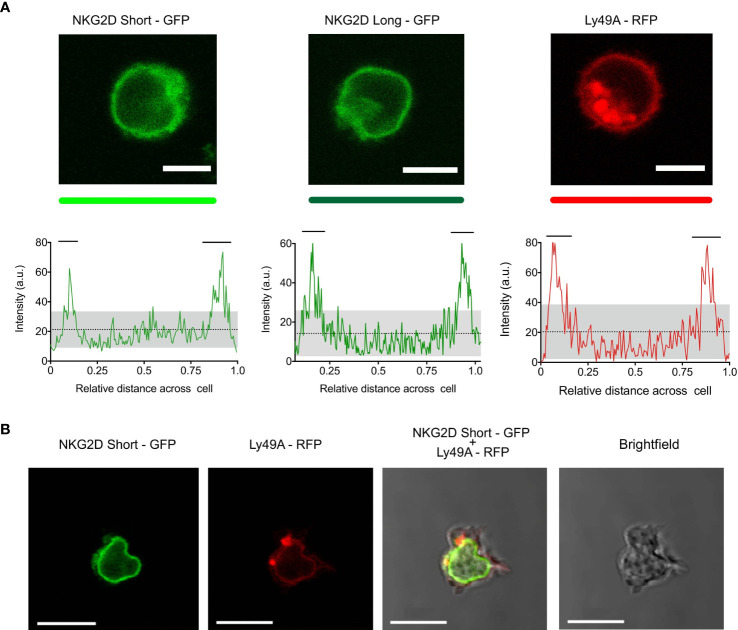
Primary natural killer (NK) cells express NKG2D-GFP and Ly49A-RFP receptors at the cell surface. **(A)** Confocal z-stack live cell imaging of NKG2D short-GFP, NKG2D long-GFP, and Ly49A-RFP splenic NK cells obtained from retrogenic mice (top). The plot profiles show GFP and RFP signal intensities across the cell diameter (bottom). The mean signal intensity is represented by a dotted line. Gray areas represent the lower and upper 95%CI of the mean. Horizontal bars indicate areas of the plot where the signal is above the upper 95%CI of the mean. One representative experiment is shown. Scale bars, 5 um. **(B)** Confocal z-stack live cell imaging of double-transduced NKG2D short-GFP + Ly49A-RFP NK cell (*in vivo*). Scale bars, 10 um.

Both NKG2D-GFP and Ly49A-RFP were expressed at the cell surface (**Figures 2A, B**), and RFP and GFP intensities were correlated with the cell surface expression levels of Ly49A and NKG2D, respectively (**Figure 1C**). The cell surface expression of NKG2D isoforms varied between CD8+ T cells and NK cells, where we observed that the relative percentage of NKG2D-expressing cells was higher on NK cells than CD8+ T cell hybridomas. NKG2D-S-GFP presented higher levels of expression when compared with NKG2D-L-GFP (**Supplementary Figure S6**). This is consistent with the results obtained for NKG2D in previous studies (36–38). We successfully generated NK cells expressing FP-tagged receptors in B6 mice using the RT mice technique (**Figures 1**, **2**). NK cells expressing NKG2D-GFP and Ly49A-RFP were detected in murine peripheral blood and spleen for up to 4 months (**Supplementary Figure S7**). It is therefore apparent that authentic primary NK cells expressing FP-tagged NK cell receptors can be generated using the RT mice technique. This establishes proof-of-principle for applying this technique to produce NK cells expressing chimeric receptors. Our results also indicate that FP-tagging of NKG2D and Ly49A does not significantly impair their cell surface expression, providing compelling evidence for the application of this technique in colocalization studies.

### NKG2D-GFP Associates With Adaptor Proteins DAP10 and DAP12

**Figure 3:**
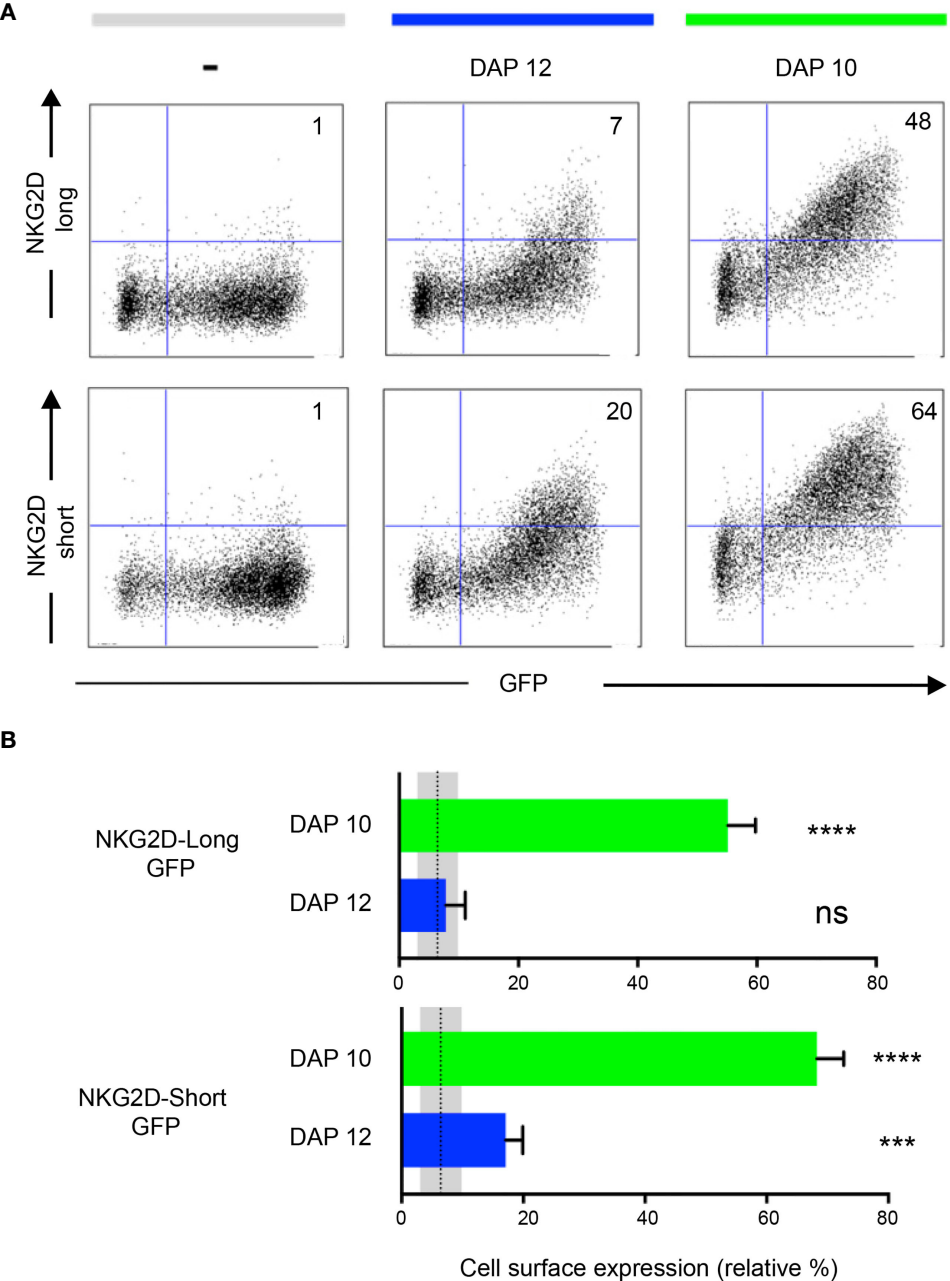
NKG2D-GFP associates with adaptor proteins DAP10 and DAP12. **(A)** Expression of NKG2D-GFP short and long isoforms at the cell surface depends on the association with DAP10 or DAP12. Each NKG2D forms a homodimer, and each assembles with a DAP10/12 homodimer. Both NKG2D and Ly49A are type II membrane proteins (*i*.*e*., N terminus intracellularly and C terminus extracellularly). HEK 293T cells were transiently co-transfected with pTagGFP-NKG2D short or long isoform and either DAP12 or DAP10 as indicated. The cells were analyzed by flow cytometry for NKG2D surface expression. The numbers indicate the percentage of cells in the respective quadrant. One representative experiment is shown. **(B)** NKG2D-GFP short isoform associates with both DAP10 and DAP12. Both isoforms can associate with DAP10, but DAP12 associates significantly better with the short than with the long NKG2D-GFP isoform. The bar graphs show the mean percentage ± SD from four replicates, and the bars with a statistically significant difference are shown as follows: *** p<0.001, **** p<0.0001. ns, no statistically significant difference (p>0.05). Unpaired *T*-tests were performed to assess statistical significance in comparison with the untransfected control. The mean is represented by a dotted line, and the gray areas represent the mean percentage ± SD of untransfected control (-). The data are representative of three separate experiments.

It is known that the NKG2D-S isoform associates with both DAP10 and DAP12, whereas the NKG2D-L isoform associates only with DAP10. Each NKG2D forms a homodimer, and each assembles with a DAP10/12 homodimer. Both NKG2D and Ly49A are type II membrane proteins (*i*.*e*., N terminus intracellularly and C terminus extracellularly; **Figure 1**). NKG2D expression is dependent on DAP10 or DAP12 association. DAP10-associated NKG2D isoforms are present in both NK and activated CD8+T cells. DAP12 association with the NKG2D short isoform occurs in NK cells only (36–38). Here we tested the effect of GFP tagging on both NKG2D isoforms and their association with DAP10 and 12. Different NKG2D-GFP isoforms were found to associate with either DAP10 or DAP12 (**Figure 3A**). The relative percentage of NKG2D+/GFP+ cells was analyzed (**Figure 3B**). NKG2D-L-GFP was found to associate with DAP10 (*P* < 0.0001) but not with DAP12 (*P* = 0.6232), whereas NKG2D-S-GFP was found to associate with both, but more efficiently with DAP10 (*P* < 0.0001) than DAP12 (*P* = 0.0009). Thus, NKG2D-S-GFP was generally found to associate more efficiently with both adaptor proteins than NKG2D-L-GFP. These differences in adaptor association are consistent with previous studies showing that the cell surface expression of NKG2D requires stabilization by DAP10 or DAP12 molecules (36). However, it is possible that the tagging of GFP onto NKG2D-S may affect its association with DAP12. This would be consistent with the hypothesis that elongating the NKG2D cytoplasmic tail affects DAP12 association, as suggested previously (38). Thus, it seems that DAP12 association is more dependent on the length of the NKG2D cytoplasmic tail than DAP10. NKG2D-GFP signaling and functionality were not directly tested. Nevertheless, NKG2D-GFP association with DAP10 and DAP12 likely indicates maintenance of functionality. Functionality has also been previously reported for human NKG2D-L fused to GFP (31).

### Activating and Inhibitory Ligand Dimensions Affect NK Cell Responses

**Figure 4:**
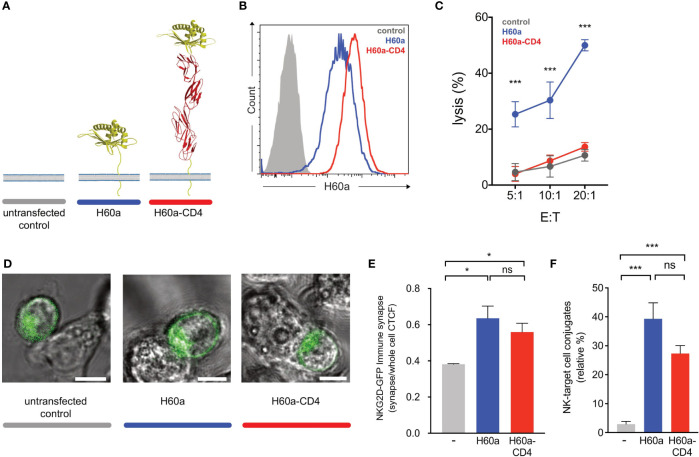
Activating ligand dimensions affect natural killer (NK) cell activation. **(A)** Schematic representation of H60a NKG2D ligand and its elongated form. The insertion of four IgSF domains creates a predicted elongation of 12 nm. For illustrative purposes, the RAE-1B structure was used to represent H60a. **(B)** H60 cell surface staining of RMA transfectants (antibody clone 205326, R&D Systems). The control (solid gray) corresponds to untransfected RMA cells; H60a (blue) and H60a-CD4 (red) correspond to RMA clones with similar levels of ligand expression. **(C)** Elongation of H60a reduces NK cell activation. Untransfected RMA (control, gray) and RMA H60a (H60a, blue), and RMA H60a-CD4 (H60a CD4, red) transfected cells expressing similar levels of ligands were used as target cells. IL-2-expanded NK cells from C57BL/6 mice were used in a killing assay at the indicated effector/target (*E*:*T*) ratios. Data show the mean ± SD %lysis (n = 3), and the groups with a statistically significant difference are shown as follows: *p < 0.05, ***p < 0.001; ns, no statistically significant difference (p > 0.05). Multiple unpaired *t*-tests were used by applying the Holm–Sidak method. Data are representative of two independent experiments. **(D)** Confocal z-stack live cell imaging of NKG2D short-GFP NK cells *ex vivo* by targeting untransfected, H60a, or H60a-CD4-expressing RMA cells. **(E)** Elongated H60a supports efficient target cell conjugate formation with NK cells and NKG2D-GFP accumulation at the immune synapse. **(F)** NK–target cell conjugates are formed between NK cells and H60a and H60a-CD4 target cells with no significant difference between both target cells. The data are representative of 3 separate experiments. Untransfected RMA cells (gray), H60a-expressing RMA cells (blue), and H60a-CD4-expressing RMA cells (red). CTCF, corrected total cell fluorescence.

**Figure 5:**
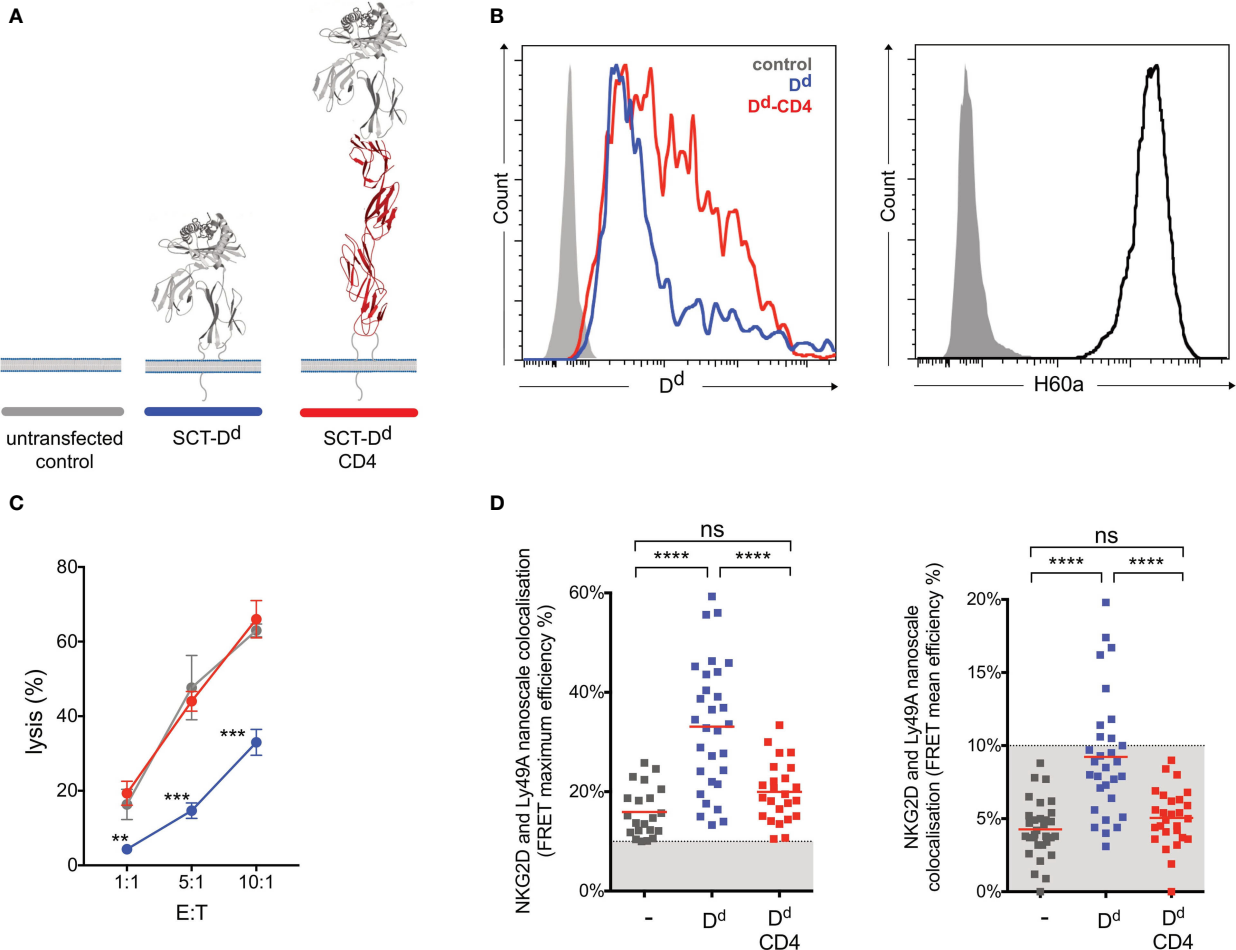
Ly49A-mediated inhibition of NKG2D-mediated natural killer (NK) cell activation correlates with the colocalization of Ly49A with NKG2D at the nanometer scale. **(A)** Schematic representation of SCT Dd Ly49A ligand and its elongated form. **(B)** H-2-Dd cell surface staining (antibody clone 34-2-12) of NIH3T3 transfectants (left panel). The control (solid gray) corresponds to untransfected NIH3T3 cells, while Dd (blue) and Dd-CD4 (red) correspond to NIH3T3 cells with similar levels of ligand expression. NIH3T3 cells endogenously express H60a (right panel). **(C)** Elongation of Dd reduces Ly49A-mediated NK cell inhibition. Untransfected NIH3T3 (control, grey), NIH3T3 SCT Dd (Dd, blue), and NIH3T3 SCT Dd-CD4 (Dd-CD4, red) transfected cells expressing similar levels of ligands were used as target cells. IL-2-expanded NK cells from C57BL/6 mice were used in a killing assay at the indicated effector/target (*E*:*T*) ratios. Data show the mean ± SD %lysis (*n* = 3), and groups with a statistically significant difference are shown as follows: **p < 0.01, ***p < 0.001, ****p < 0.0001; ns, no statistically significant difference (*p* > 0.05). Two-way analysis of variance (ANOVA) with *p* = 0.0015 and multiple unpaired *t*-tests were used by applying the Holm–Sidak method. Data are representative of two independent experiments. **(D)** Colocalization of NKG2D and Ly49A at the nanometer scale correlates with NK cell signal integration. Similarly, lower average and maximum FRET values were observed for NK immune synapses obtained using untransfected NIH3T3 or NIH3T3 SCT Dd-CD4 transfected cells. Higher levels of NKG2D and Ly49A colocalization were observed using NIH3T3 SCT Dd transfected cells. FRET efficiency was calculated pixel by pixel, and the mean and maximum values registered for each NK immune synapse (IS) are shown in the right and left panels, respectively. Each square point represents a unique NK synapse. All NK ISs were acquired using NK cells expressing both NKG2D-GFP and Ly49A-RFP. The results are from three independent experiments using primary NK cells harvested from 3 different retrogenic mice. A minimum threshold of 10% FRET efficiency was applied. The graphs show the mean percentage ± SEM from at least 26 synapses. Ordinary one-way ANOVA and Tukey’s multiple-comparisons test were used, and groups with a statistically significant difference are shown as follows: **p < 0.01, ***p < 0.001, ****p < 0.0001; ns, no statistical significance (*p* > 0.05).

Earlier studies showed that elongated NK cell ligands affect NK cell responses, with elongation of activating and inhibitory ligands affecting NK cell activation and inhibition, respectively (14). In this study, two NK cell ligands were used to test whether activating and inhibitory ligand dimensions affect NK cell responses. Activating, H60a-CD4, and inhibitory, SCT Dd-CD4, elongated ligands were generated by the insertion of four inert human IgSF CD4 domains. Here we demonstrate that elongating H60a weakens NK cell activation and elongating Dd reduces NK cell inhibition. These results are consistent with previous studies (14, 15). RMA target cells were transfected with H60a and H60a-CD4 (**Figure 4A**). Both constructs were found to be expressed on RMA cells with a similar cell surface expression (**Figure 4B**). Elongation of H60a was observed to reduce NK cell lysis at different effector/target (*E*:*T*) cell ratios (5:1, *P* = 0.002; 10:1, *P* = 0.005; 20:1, *P* < 0.0001; **Figure 4C**). These results were found to be NKG2D dependent (data not shown). Thus, it is evident in our study that elongated activating NK cell ligands have a detrimental effect on NK cell activation. Next, we investigated whether the elongation of H60a interfered with NKG2D binding and cell–cell conjugate formation. No accumulation of NKG2D at the immune synapse was observed with untransfected RMA cells (**Figure 4D**). NKG2D accumulation was found to increase at the immune synapse formed by RMA cells expressing either H60a (*P* = 0.0148) or H60a-CD4 (*P* = 0.0162; **Figures 4D, E**). A similar observation was made for cell–cell conjugation; NK cells were found not to form conjugates efficiently with untransfected RMA cells, but they formed conjugates with RMA cells expressing H60a (*P* < 0.001) or elongated H60a-CD4 (*P* < 0.001; **Figure 4F**). Our results confirm that the elongation of H60a affects NK cell activation but not by impairing NKG2D binding and cell–cell conjugation. Next, we investigated whether ligand dimensions play a role in NK cell inhibition. **Figure 5** presents the impact of the elongation of SCT Dd in NK cell inhibition. NIH3T3 cells transfected with SCT Dd and SCT Dd-CD4 ligands were used as target cells (**Figure 5A**). Both constructs were expressed on NIH3T3 cells at similar levels (**Figure 5B**, left). SCT Dd and SCT Dd-CD4 molecules were also efficiently transfected and correctly folded on CHO cell surface as demonstrated by antibody staining with two different anti H2-Dd monoclonal antibodies (**Supplementary Figure S8**). NIH3T3 cells were observed to endogenously express the NKG2D ligand, H60a (**Figure 5B**, right). NIH3T3 cells expressing SCT Dd were more resistant to NK cell lysis by Ly49A+-enriched NK cells at different *E*:*T* ratios (1:1, *P* = 0.007; 5:1, *P* = 0.003; 10:1, *P* = 0.0002; **Figure 5C**). However, the elongation of the Ly49A ligand SCT Dd was found to reduce Ly49A-mediated inhibition. Using the elongated SCT Dd ligand, lysis was higher and equivalent to the levels of the NIH3T3 untransfected control. These results were in agreement with a loss of function of the elongated ligand also observed for NK cell activation when using H60a-CD4 ligand (see **Figure 4**). Thus, it is evident that inhibitory NK cell ligand dimensions control NK cell responses, in agreement with previous observations (14, 15). The differences in NK cell inhibition are unlikely to be due to a different ligand expression since the levels of ligand expression were similar (**Figure 5B**). It has been reported that elongated ligands can increase the intermembrane space in the IS (12). Thus, it was hypothesized that ligand elongation affects the nanoscale colocalization of receptors.

### Nanoscale Colocalization of NKG2D and Ly49A Correlates With NK Cell Inhibition

We next tested whether NKG2D and Ly49A colocalize at the nanometer scale upon IS formation using different target cells (**Figure 5**). We reasoned that Dd elongation could abrogate relative NKG2D and Ly49A colocalization, thereby impacting NK cell signal integration. To investigate this question, we used a natural immune synapse model using NIH3T3 target cells. This strategy allows for the visualization of the NK IS *en face* in a single z-stack plane. Due to their morphology and adherence, NIH3T3 cells produce a monolayer of target cells and a large surface area for NK cell interaction (39–41). The use of a monolayer of NIH3T3 cells is a novel experimental approach to study the NK IS. NIH3T3 cells present a small height and thickness as revealed by atomic force microscopy studies (42). This fact makes NIH3T3 a promising cell line to use in NK IS studies. We expressed a normal-length and an elongated form of Dd in NIH3T3 cells, which also constitutively express H60a. In this study, the H2-Dd MHC-I molecule was expressed as a single-chain trimer. The H2-Dd SCT construct was previously generated by an overlap PCR of the Dd heavy chain and an existing H2-Kb SCT construct, followed by a mutagenesis reaction to change the peptide sequence to P18, a decapeptide from the HIV-1 envelope glycoprotein V3 loop (14). H2-Dd SCT extended version has previously been expressed in CHO cells and shown to be efficiently expressed and folded correctly at the cell surface (**Supplementary Figure S8**). ISs were visualized by confocal microscopy using NKG2D-GFP and Ly49A-RFP-expressing splenic NK cells. Authentic primary NK cells from RT mice were used. We used FRET as an accurate nanoscale measure of distance between NKG2D and Ly49A upon IS formation. NKG2D and Ly49A nanometer-scale colocalization was found to vary according to ligand expression and dimensions (**Figure 5D**). Only in the presence of both NK cell ligands, H60a and Dd, do NKG2D and Ly49A colocalize at a nanometer scale, and Ly49A-dependent inhibition occurs. Interestingly, increasing the size of the Ly49A ligand was shown to affect its nanoscale organization at the IS, decreasing the nanoscale colocalization with NKG2D. The mean and maximum FRET efficiency values were analyzed for each synapse, as well as the total FRET area. All values were measured using the acceptor photobleaching FRET technique (**Supplementary Figure S9**). The FRET mean efficiency ranged between 0 and 20%. In this study, FRET values below 10% efficiency can be hypothetically associated with background noise due to the inherent characteristics of the experimental approach. Therefore, we defined a minimum threshold of 10% FRET efficiency. Our results showed that NKG2D and Ly49A colocalize in the presence of both ligands (blue, *P* < 0.0001, *n* = 30; **Figure 5D**). Interestingly, in comparison with SCT Dd, Ly49A was found to segregate from NKG2D in the presence of the Ly49A elongated ligand, SCT Dd-CD4 (red, *P* < 0.0001, *n* = 27; **Figure 5D**). NKG2D and Ly49A colocalization did not occur in the absence of SCT Dd or SCT Dd-CD4 (gray, *n* = 31; **Figure 5D**). Similar results were also observed for each NKG2D isoform. Interestingly, NKG2D long isoform showed some FRET compared to the untransfected control in the presence of SCT Dd-CD4. This can be due to a weak binding and inhibitory synapse formation of SCT Dd-CD4 and Ly49A. The length differences between NKG2D short and long isoforms can also potentially cause changes in DAP10/12 association and respective NKG2D cell surface that may slightly impact the FRET values in colocalization with Ly49A-RFP. However, the difference observed between maximum FRET efficiency values was less than the difference between the SCT Dd and SCT Dd-CD4 synapses (**Supplementary Figures S10, S11**). Thus, Ly49A-mediated inhibition of NKG2D occurred when Ly49A was colocalized with NKG2D, correlating with NKG2D and Ly49A functional signal integration (**Figures 5C, D**). These results show that elongating NK cell ligands impact their nanoscale colocalization.

## Discussion

Previous studies have demonstrated that NK cell and T cell ligand dimensions control the immune cell responses (12–14). We hypothesized that this phenomenon occurs due to a differential nanoscale colocalization of receptors at the IS based on ectodomain size, as initially suggested by the K-S model (11). However, it is challenging to study the NK IS organization of receptors at the nanometer scale. Before the emergence of super-resolution (SR) microscopy, NK IS studies focused mainly on the micrometer-scale organization of receptors, *i*.*e*., microclusters (31, 43, 44). Following the development of SR microscopy, we are now starting to have an insight of the nanometer-scale organization of immune receptors at the cell surface (45, 46). However, the use of SR techniques poses some challenges, *e*.*g*., poor image quality or artifacts commonly introduced during sample preparation (47). Besides the limits of SR microscopy sample preparation, it is also challenging to use a reproducible NK IS model while preserving its physiological characteristics. Thus, previous studies have used NK cell-like lines and artificial target cell surfaces, *e*.*g*., lipid bilayers or cross-linking antibody surfaces to study the NK IS (22, 31, 43, 48). However, neither the use of cell lines or lipid bilayers or antibody-coated surfaces is ideal to reproduce the exact molecular conditions observed in a *bona fide* NK IS cell–cell interface. Therefore, in this study, we used primary NK cells from RT mice and a monolayer of NIH3T3 target cells to image the cell–cell interface of an authentic NK IS. Our aim was to measure the nanoscale colocalization by FRET between two NK cell receptors inside a physiological NK IS. Primary NK cells expressing NKG2D-GFP (short and long isoforms) and Ly49A-RFP were generated, and both FP-tagged NK cell receptors were successfully expressed at the cell surface *in vivo*. The use of primary NK cells from RT mice constitutes a novel approach to generate chimeric receptors expressed in NK cells. However, it should be noted that the use of RT mice also has some disadvantages, in particular, (i) the low number of NK cells expressing both tagged receptors, (ii) new mice need to be produced each time, with each mouse being a new and single-use founder (35), and (iii) the off-target expression of NK cell receptors on other immune cell lineages. Future work could use a CRISPR/Cas or a Ncr1-Cre mouse model to overcome these disadvantages (49). Nevertheless, the use of authentic, primary NK cells *ex vivo* represents an improvement over the use of NK cell lines to study the NK IS. Our results demonstrate that, in a prototypical inhibitory synapse in the presence of both activating and inhibitory ligands, NKG2D and Ly49A colocalize at the nanometer scale. However, in the presence of NKG2D ligands only, NKG2D and Ly49A do not colocalize. Interestingly, increasing the size of the Ly49A ligand decreased its nanoscale colocalization with NKG2D. Moreover, the extension of Ly49A ligand correlated with an impairment of Ly49A-mediated inhibition. NKG2D was found to accumulate at the IS similarly well with target cells expressing the elongated H60a-CD4 as with H60a. The H60a-CD4 construct also promotes cell–cell conjugation by interacting with NKG2D. Thus, ligand elongation did not cause a significant disruption in receptor binding or NK IS formation to explain the functional differences observed. In fact, the introduction of two, three, or four (CD4) IgSf domains in the extracellular domains has been previously established and used to study the molecular mechanism of TCR triggering. This molecular elongation leads to a gradual decrease of TCR activation, proportional to the SCT MHC-I extension. A similar observation was made also using elongated NK cell activating receptors (12–14). A non-specific interaction of the four IgSF (CD4) with itself is unlikely since the insertion of the IgSF ectodomains did not affect SCT Dd folding or cell surface expression (**Supplementary Figure S8**). Another fact to consider is the possible relative bending of the elongated ligand, but since the IgSF domains are known to be very rigid, a significant bending is unlikely. Moreover, the introduction of the IgSF domains should not cause intra- or inter-specific interactions, as they are functionally inert and relatively widespread in most immune receptors. Electron microscopy studies have also reported that the elongated ligands impose a small but significant increase in the intermembrane spacing in the IS, indicating elongation (12). In our study, we did not explore the possibility that crosslinking the CD4 spaced molecules with an anti-CD4 antibody or CD4 binding protein might counteract or interfere with the elongation effect and relative clustering or colocalization. This possibility would be a fascinating hypothesis to test in the future and further widen our understanding of the elongation effect. However, it would also be challenging to fully assess the degree of molecular modifications caused by such changes in an isolated way. Therefore, we hypothesize that NK signal integration occurs by the relative size-dependent colocalization of receptors on the NK IS, and this is the explanation for the functional differences observed for elongated ligands. Considering the differences observed at the nanometer scale, our results further support recent evidence that immune cell receptors show a nanometer-scale organization, with function-associated implications (19, 22, 48, 50–53). Interestingly, there is mounting evidence that the molecular mechanisms behind immune inhibition are, in part, spatially restricted and common to various immune cells, such as T, B, and NK cells (54–58). Thus, it is hypothesized that net phosphorylation occurs due to the proximity of inhibitory receptors to activating receptors. SHP-1/2 phosphatases are known to become activated when bound to phosphorylated ITIMs. This binding releases the phosphatase domain, making it available to dephosphorylate proximal molecules (58). Thus, dephosphorylation is expected to occur in proximity to the inhibitory receptors. The data presented in this study are consistent with this hypothesis. Our results demonstrate a correlation between receptor colocalization, signal integration, and effector responses. Our results provide compelling evidence that the colocalization of Ly49A with NKG2D is key to the Ly49A-mediated inhibitory mechanism. We also showed that disruption of colocalization by elongating Ly49A disrupted inhibition. Thus, our results show that small changes in the nanoscale organization of receptors have significant consequences on NK cell responses. Small differences in ligand ectodomain size seem to impair functionality by decreasing colocalization at the nanometer scale. Our observations are consistent with the results obtained in previous studies and consistent with the K-S model (11). Thus, it is evident that signal integration is not only determined by the levels of expression of receptors at the cell surface but also by the nanoscale organization of receptors. In conclusion, the nanoscale contact points at the IS may determine the balance of signals in NK cells. The nanometer-scale changes of colocalization of receptors may tip the phosphorylation balance between kinases and phosphatases and control the NK cell signal integration outcome. Following the observation on the nanoscale colocalization of Ly49A and NKG2D upon NK IS formation, we postulate that the activation threshold, balance, and integration of NK cell signals depend on the relative colocalization of activating and inhibitory NK cell receptors at the IS.

## Data Availability Statement

The original contributions presented in the study are included in the article/**Supplementary Material**. Further inquiries can be directed to the corresponding author.

## Ethics Statement

The animal study was reviewed and approved by Imperial College London as well as approved by the Institutional Animal Welfare and Ethical Review Committee and by the Home Office.

## Author Contributions

DT performed all the experiments, DT and KG conceived the study, designed the experiments, and analyzed the data. RH and PMP helped in designing, performing, and analyzing the experiments involving confocal microscopy and FRET. NG helped in designing, performing, and analyzing the experiments involving *Klrk1* -/- mice. JD helped in designing, performing, and analyzing the experiments using the retrogenic mice technique. DT wrote the paper. All authors contributed to the article and approved the submitted version.

## Conflict of Interest

The authors declare that the research was conducted in the absence of any commercial or financial relationships that could be construed as a potential conflict of interest.

## Publisher’s Note

All claims expressed in this article are solely those of the authors and do not necessarily represent those of their affiliated organizations, or those of the publisher, the editors and the reviewers. Any product that may be evaluated in this article, or claim that may be made by its manufacturer, is not guaranteed or endorsed by the publisher.
