# Supplementary material for: Nanoscale Colocalization of NK Cell Activating and Inhibitory Receptors Controls Signal Integration
Source: Front Immunol. 2022 Jun 1;13:868496. doi: 10.3389/fimmu.2022.868496 (PMC9198454; doi:10.3389/fimmu.2022.868496)
Supplement: Supplementary file 1 [file DataSheet_1.docx]

# Supplementary Material

**
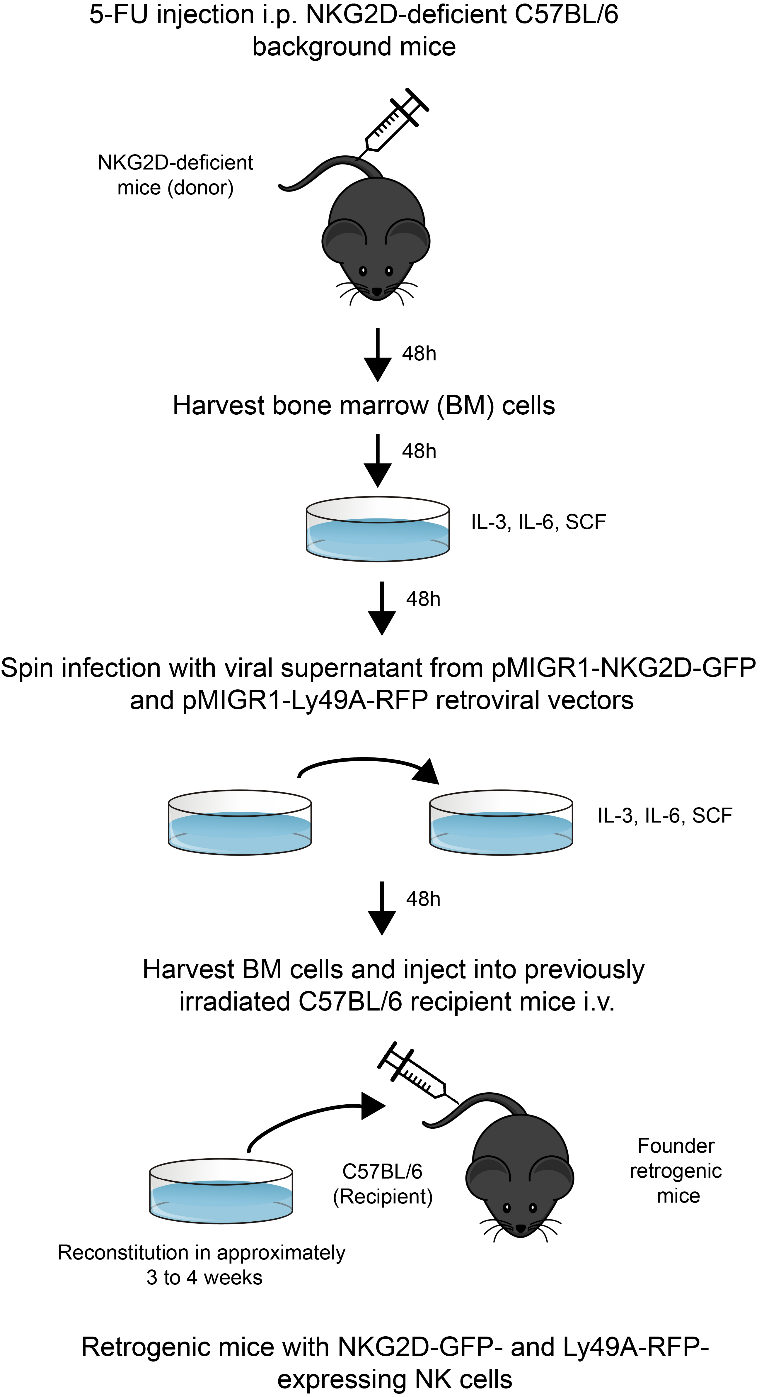
Figure supplementary 1 - Schematic representation of the retrogenic (RT) mice generation using NKG2D-deficient mice**

**Figure S1** - The retrogenic mice technique applied both pMIGR1-NKG2D-GFP (both isoforms) and pMIGR1-Ly49A-RFP retroviral vectors to transduce HPCs from Klrk1-/- C57BL/6 mice. NK cells expressing FP-tagged NK cell receptors can be harvested approximately 2 to 3 weeks following retroviral-mediated stem cell gene transfer. 5-FU, 5-fluorouracil; IL- 3, interleukin-3; i.p., intra-peritoneal; i.v., intravenous; SCF, stem cell factor.}

**Figure supplementary 2 - 293T cells were efficiently transfected with pMIGR1-NKG2D-GFP**


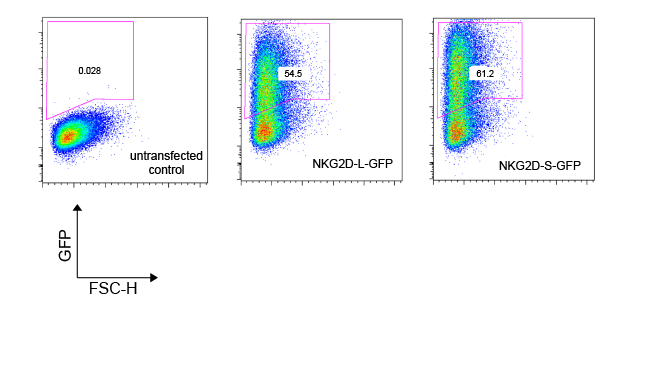


Figure S2 - 293T Phoenix ecotropic cells were used as packaging cell line for the production of retroviral particles expressing NKG2D-GFP. As expected, transfected 293T cells showed a high efficiency of transfection, indicated by the GFP+ population for both pMIGR1-NKG2D short-GFP and pMIGR1-NKG2D long-GFP. GFP+ signal is represented in the Y axis and forward side scatter (height) in the X axis. Numbers indicate the percentage of cells in the respective gating. A single experiment, representative of more than 30 separate experiments, is shown.}

**Figure supplementary 3 - 293T cells were efficiently transfected with pMIGR1-Ly49A-RFP**


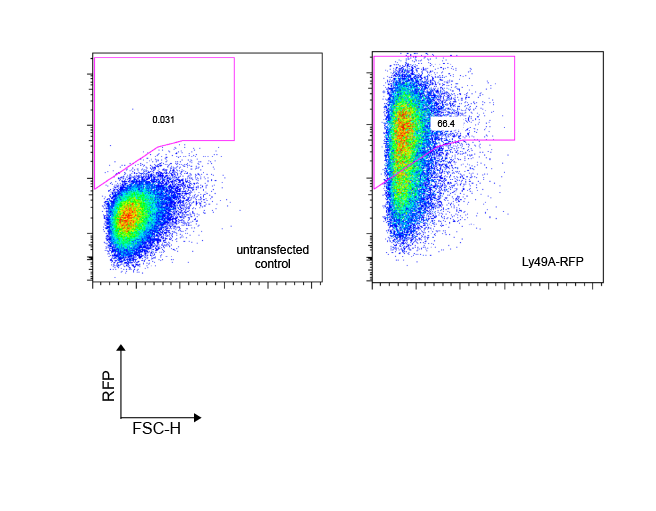


Figure S3 - 293T Phoenix ecotropic cells showed a high efficiency of transfection, indicated by the RFP+ population representative of Ly49A-RFP receptors produced. RFP signal is represented on the Y axis and forward side scatter (height) on the X axis. Numbers indicate the percentage of cells in the respective gating. A single experiment is shown, representative of over 30 separate experiments.

**Figure supplementary 4 - NKG2D deficient mice were used as BM cell donors**

**
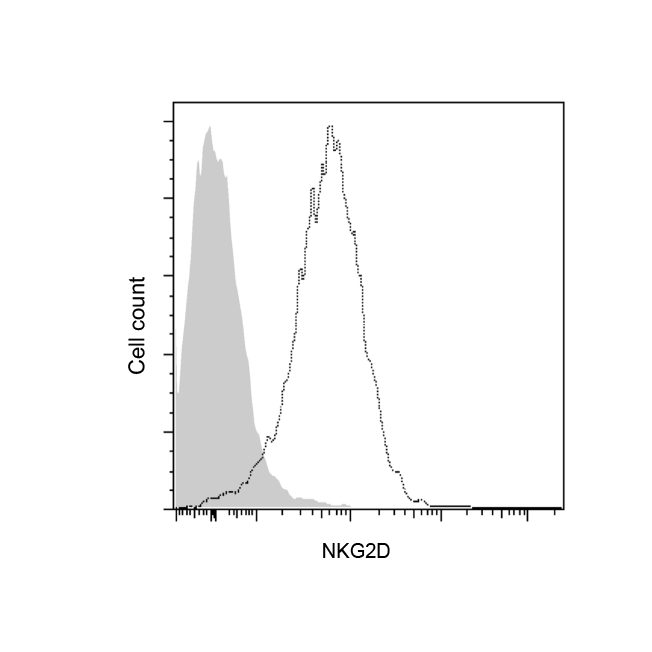
**

Figure S4 - Flow cytometry analysis of NKG2D expression on spleen NK cells, CD3-, CD8-, NK1.1+ and DX5+ NK cells of C57BL/6 (dotted line) and NKG2D-deficient (Klrk1-/-) retrogenic (RT) mice (grey area) is shown. RT donor mice NK cells (grey area) were confirmed to not express NKG2D.

**Figure supplementary 5 - HPCs were transduced with both NKG2D-GFP and Ly49A-RFP**

**
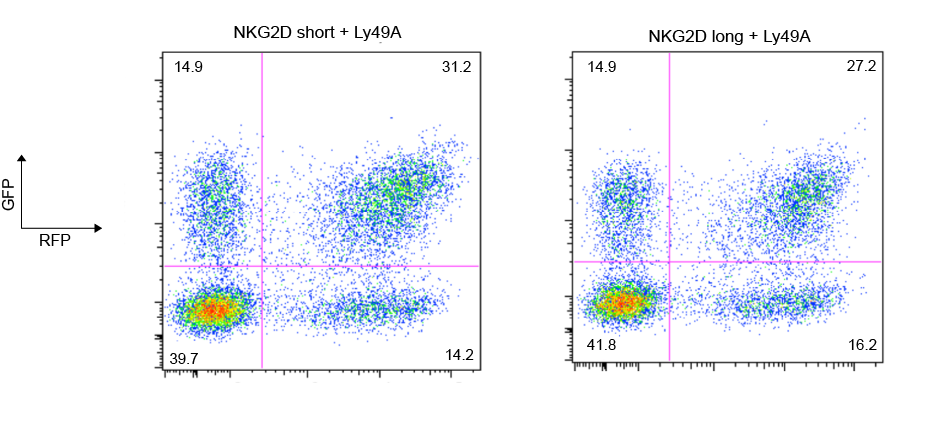
**

Figure S5 - The plots represent double transduction experiments, in which two separate viral supernatants from pMIGR1-NKG2D-GFP and pMIGR1-Ly49A-RFP were used simultaneously to obtain doubly transduced HPCs (NKG2D short and long isoforms, left and right, respectively). These experiments were performed using HPCs from NKG2D-deficient C57BL/6 donor mice. Numbers indicate the percentage of cells in the respective quadrant. The data are representative of more than 3 separate experiments.

**Figure supplementary 6 - Relative percentage of NKG2D-GFP and Ly49A-RFP cell surface expression on CD8+ T or NK cells.**


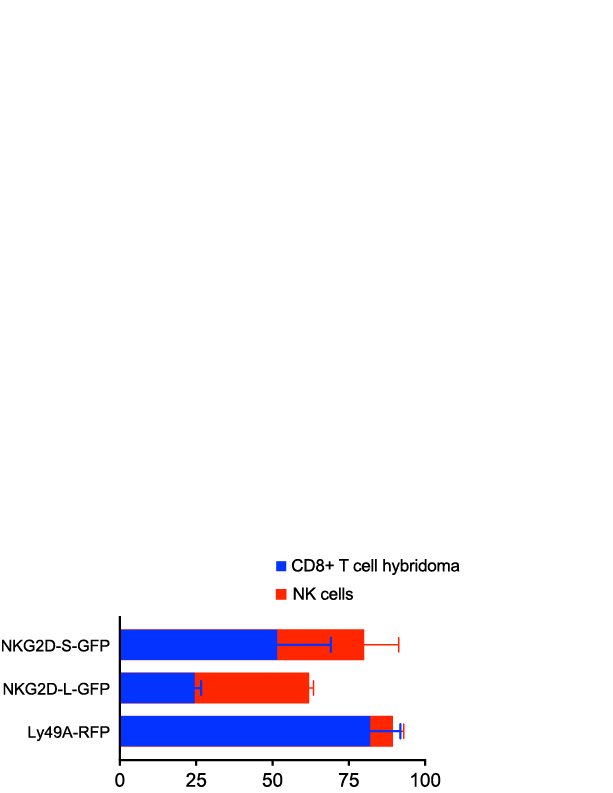


Figure S6 - NKG2D-GFP receptors are expressed in higher levels on NK cells (red) than CD8+ T cell hybridomas (green), in particular for NKG2D long-GFP. NKG2D short-GFP presents a higher cell surface expression on both CD8+T and NK cells. A significant difference of cell surface expression of NKG2D long-GFP is likely a consequence of a different capacity of this isoform to associate with DAP10 and DAP12.

**Figure supplementary 7 - Primary NK cells expressing NKG2D-GFP and Ly49A-RFP are efficiently generated in vivo.**


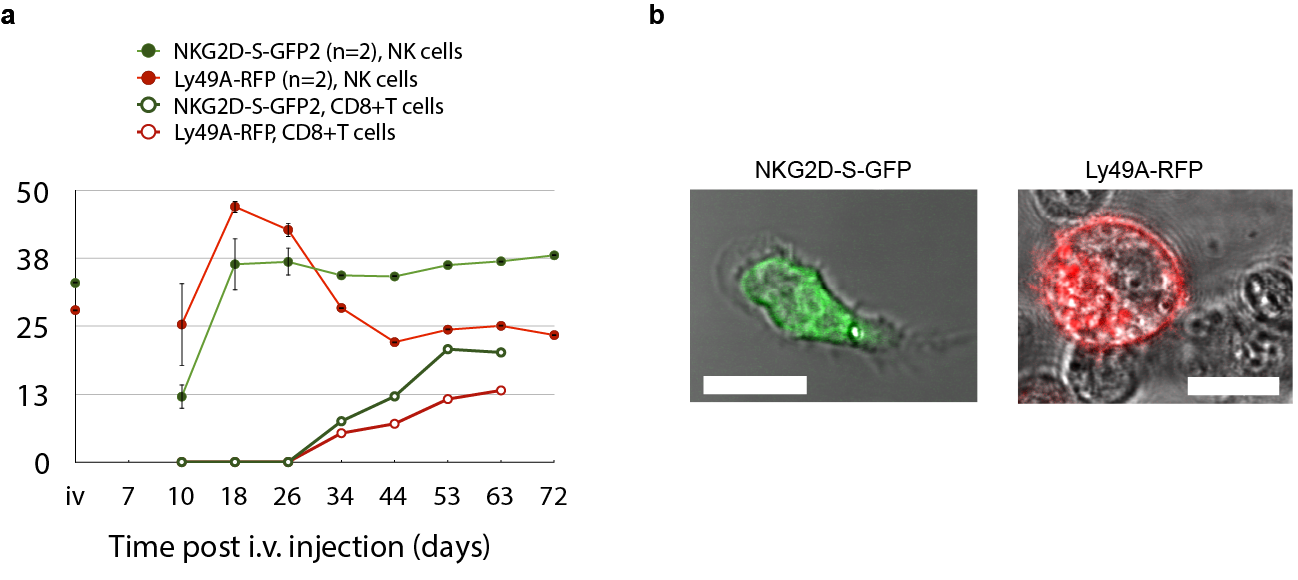


Figure S7 - **a**, Graphic timeline of the relative percentage of NKG2D-GFP- (green) and Ly49A-RFP-(red) expressing NK cells (closed circles) and CD8+ T cells (open circles) circulating in mice peripheral blood following intravenous (i.v.) injection (left). Data are shown as relative percentage of NK1.1+, CD49b+ and CD3– or CD3+ CD8+ cells resulting from post-injection reconstitution. Relative percentage corresponds to NK or CD8+ T cells during the initial four months following retroviral-mediated stem cell gene transfer. “iv” corresponds to the initial percentage of NKG2D-GFP or Ly49A-RFP transduced HPCs at the time of injection. Approximately 5 to 7 x 10^5 total BM cells were transplanted into sublethally irradiated WT recipient mice (mean ± SD, n=2). Time post i.v. injection is not to scale. **b**, Confocal z-stack image of an ex vivo primary NK cell expressing NKG2D-GFP (left) and Ly49A–RFP (right). Scale bar of 10 microns.

**Figure supplementary 8 - CHO cells were efficiently transfected with both SCT Dd and SCT Dd-CD4**


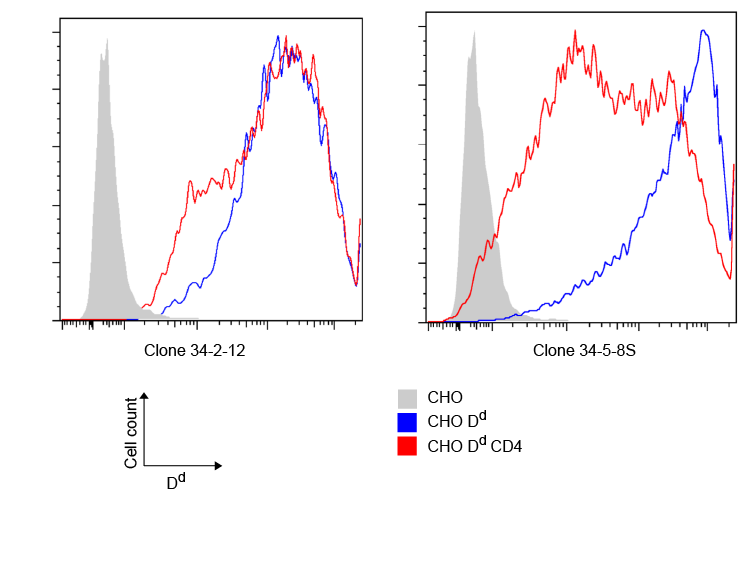


Figure S8 - CHO cells were efficiently transfected with SCT Dd or SCT Dd-CD4. Two anti-H2-Dd monoclonal antibodies (clones 34-2-12 and 34-5-8S) targeting two different H2Dd epitopes were used in order to demonstrate the correct folding and cell surface expression of both H2-Dd MHC-I single chain trimers (SCT). H2-Dd cell surface staining is represented in the X axis and cell counts in the Y axis. The control (solid grey) corresponds to untransfected CHO cells. SCT Dd (blue) and SCT Dd-CD4 (red) correspond to ligand-expressing CHO cells. One representative experiment is shown.}

**Figure supplementary 9 - Acceptor photobleaching FRET**


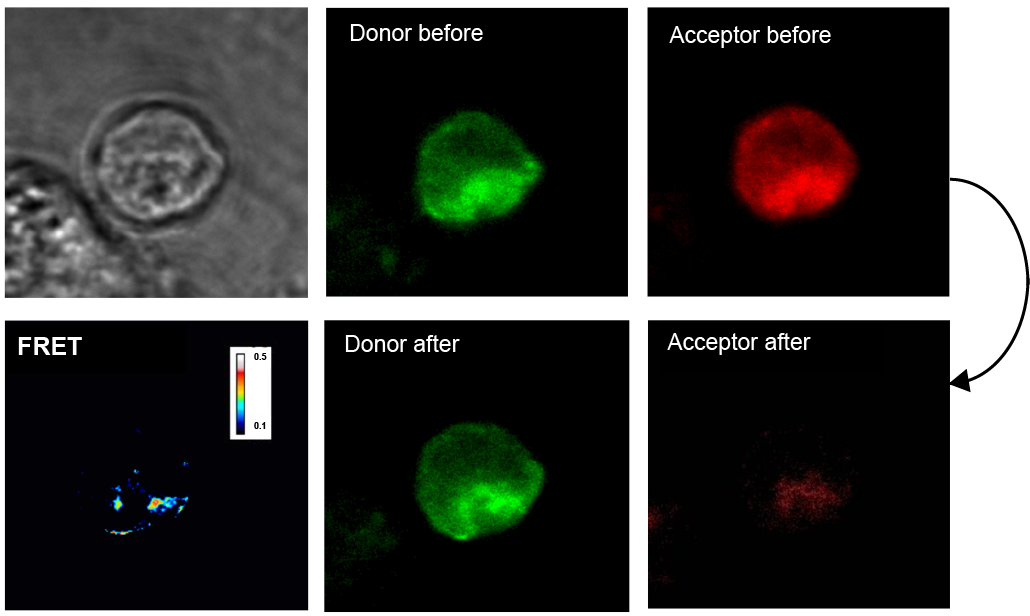


Figure S9 - A prototypical FRET acceptor photobleaching protocol is represented. This figure depicts the sequence of donor and acceptor fluorescence acquisition between an NK cell expressing NKG2D long-GFP and Ly49A-RFP in synapse with NIH3T3 cells expressing both NKG2D and Ly49A ligands. Primary splenic NK cells obtained from retrogenic mice, expressing NKG2D-GFP and Ly49A-RFP were used in these experiments. For each NK immune synapse (IS) the relative GFP (donor) and RFP (acceptor) fluorescence signals were compared before (top) and after (bottom) acceptor photobleaching. Images of GFP and RFP were acquired before (top right) and after photobleaching RFP (bottom right). The change of GFP fluorescence intensity after photobleaching was measured and FRET regions were then derived by comparing the regions of synapse where the donor (GFP) signal increased after acceptor photobleaching (bottom left image, scale bar with arbitrary units).

**Figure supplementary 10 - Maximum FRET efficiency values vary according to NK cell ligands expressed in NIH3T3 target cells in the different NK immune synapses (ISs)**

**
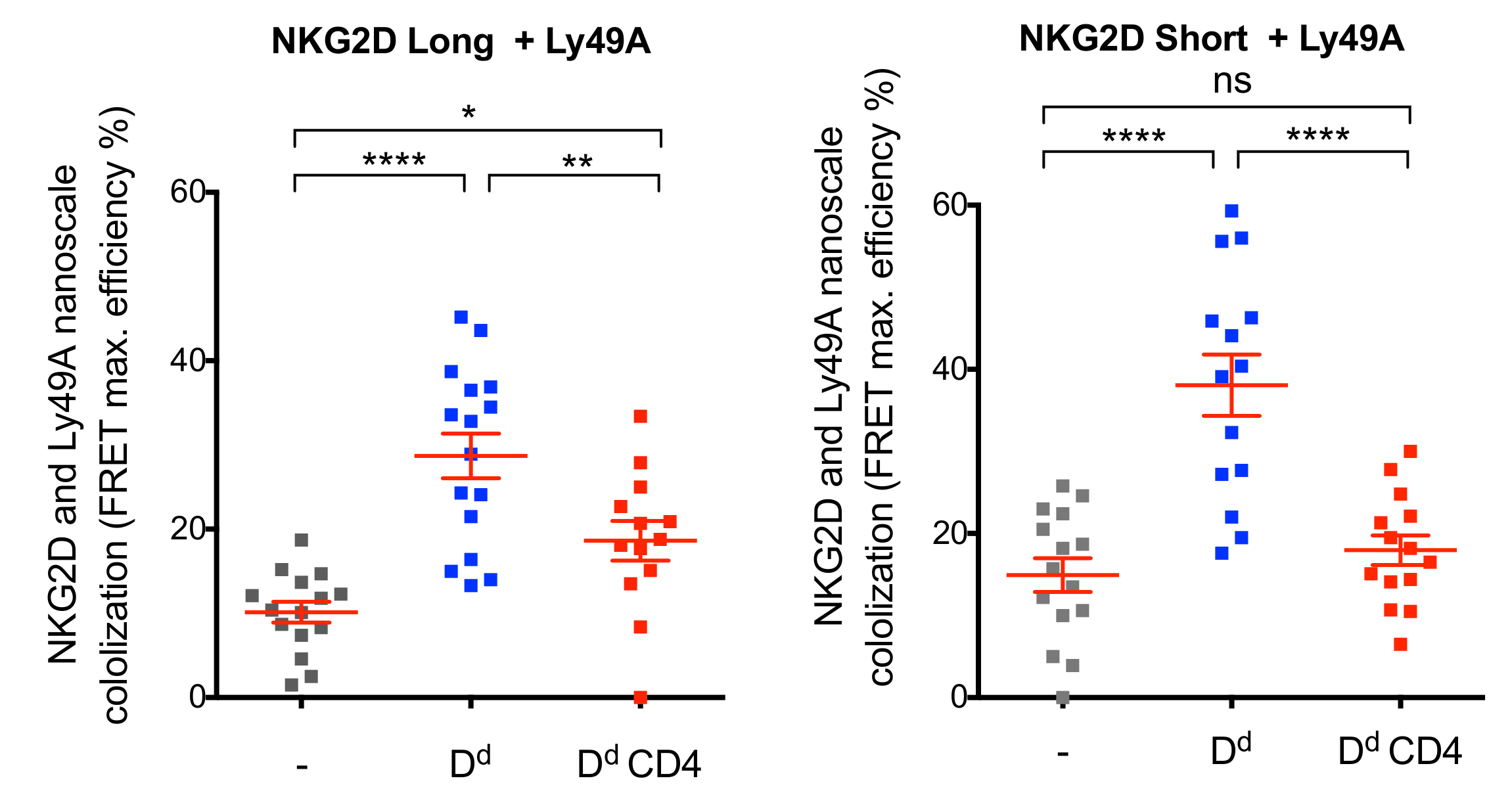
**Figure S10 - FRET efficiency was calculated pixel-by-pixel, and the maximum value registered for each NK IS is shown here. Each square point represents a unique synapse. NK ISs using NKG2D long-GFP and Ly49A-RFP-expressing NK cells are shown on the left; NK ISs using NKG2D short-GFP and Ly49A-RFP-expressing NK cells are shown on the right. These results represent NK ISs from three independent experiments, using primary NK cells harvested from at least 3 different RT mice. No minimum threshold of FRET efficiency was defined. The graphs show the mean percentage of association ± SEM from at least thirteen replicates, and groups with different statistical significance are as shown: * p<0.05, ** p<0.01, *** p<0.001, **** p<0.0001, ns, no statistical significance (p>0.05).}

**Figure supplementary 11 - Mean FRET efficiency values vary according to NK cell ligands expressed in NIH3T3 target cells in the different NK immune synapses (ISs)**

**
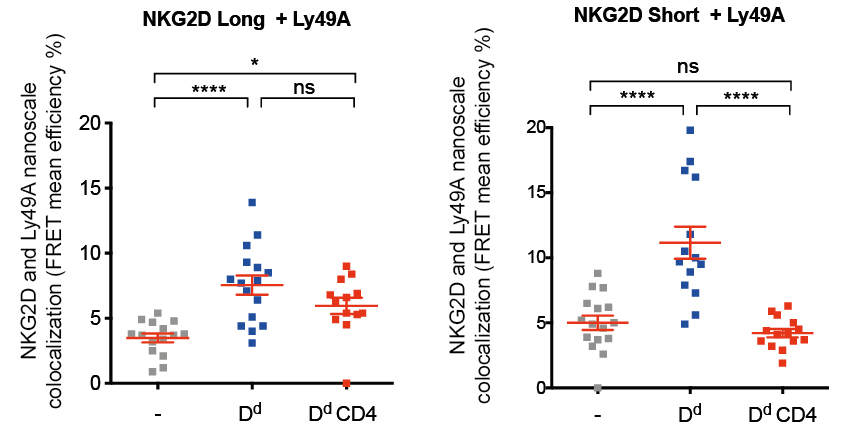
**

Figure S11 - FRET efficiency was calculated pixel-by-pixel, and the average value registered for each NK IS shown here. NK ISs using NKG2D long-GFP and Ly49A-RFP-expressing NK cells are shown on the left; NK ISs using NKG2D short-GFP and Ly49A-RFP-expressing NK cells are shown on the right. These results represent NK ISs from three independent experiments, using primary NK cells harvested from at least 3 different RT mice. No minimum threshold of FRET efficiency was defined. Each square point represents a unique NK IS. The graphs show the mean percentage of association ± SEM from at least thirteen replicates, and groups with different statistical significance are as shown: * p<0.05, ** p<0.01, *** p<0.001, **** p<0.0001, ns, no statistical significance (p>0.05).
